# SYK promotes the formation of neutrophil extracellular traps by inducing PKM2 nuclear translocation and promoting STAT3 phosphorylation to exacerbate hepatic ischemia-reperfusion injury and tumor recurrence

**DOI:** 10.1186/s10020-024-00907-7

**Published:** 2024-09-11

**Authors:** Xuejiao Chen, Chuanwei Jiang, Minhao Chen, Xiangdong Li, Wenjie Yu, Aigang Qiu, Linfeng Sun, Liyong Pu, Yuhua Shi

**Affiliations:** 1 Department of General Surgery, The Yancheng School of Clinical Medicine of Nanjing Medical University, 75 Theater Road, Yancheng, 224000 Jiangsu province China; 2 Hepatobiliary Center, The First Affiliated Hospital of Nanjing Medical University, 300 Guangzhou Road, Nanjing, 210003 Jiang Su province China

**Keywords:** Ischemia-reperfusion injury, Neutrophil Extracellular traps, Macrophage

## Abstract

**Background:**

At present, hepatic ischemia-reperfusion injury (IRI) is an important complication of partial hepatectomy and liver transplantation, and it is an important cause of poor prognosis. Spleen tyrosine kinase(SYK) plays an important role in a variety of signaling pathways in the liver, but its role in hepatic IRI is still unclear. This study aims to investigate the role and mechanism of SYK in hepatic IRI and tumor recurrence.

**Methods:**

We first observed the activation of SYK in the liver of mice in response to hepatic IRI. Subsequently, Pharmacological inhibitions of SYK were used to evaluated the effect of SYK on neutrophil recruitment and NETosis, and further explored the effect of SYK on IRI and tumor recurrence.

**Results:**

Our study shows that SYK is activated in response to hepatic IRI and aggravates liver injury. On the one hand, neutrophils SYK during the early stage of liver reperfusion increases neutrophil extracellular traps (NETs) production by promoting Pyruvate kinase M2(PKM2) nuclear translocation leading to upregulation of phosphorylated STAT3, thereby exacerbating liver inflammation and tumor recurrence. On the other hand, macrophages SYK can promote the recruitment of neutrophils and increase the activation of NLRP3 inflammasome and IL1β, which further promotes the formation of NETs.

**Conclusions:**

Our study demonstrates that neutrophil and macrophage SYK synergistically promote hepatic IRI and tumor recurrence, and SYK may be a potential target to improve postoperative hepatic IRI and tumor recurrence.

**Supplementary Information:**

The online version contains supplementary material available at 10.1186/s10020-024-00907-7.

## Background

Hepatic resection and liver transplantation are currently the most effective treatments for liver cancer. However, hepatic ischemia-reperfusion injury (IRI) during liver resection is still a great challenge and is closely related to the recurrence of liver cancer (Thorgersen et al. 2019; Nagai et al. 2015). Severe hepatic IRI after transplantation can induce acute or chronic rejection reactions, potentially leading to transplant failure (Guo et al. 2022). Therefore, it is very important to fully explore the mechanism of liver IRI and seek methods to mitigate IRI are crucial for alleviating postoperative liver injury, reducing liver cancer recurrence, and improving the success rate of transplantation. Immune activation plays a major role in the process of hepatic IR and occurs throughout the process of injury. A variety of key inflammatory components produced by immune cells in response to IR further exacerbate liver damage and activate tumor cell proliferation, adhesion, aggregation, and angiogenesis to promote liver cancer recurrence (Chen et al. 2022; Zhai et al. 2011; Li et al. 2023a, b).

Neutrophils are an important component of liver IRI and one of the first immune cells recruited to sites of injury. Activated neutrophils release neutrophil extracellular traps (NETs), which play a crucial role in triggering inflammatory responses (Kaltenmeier et al. 2022). NETs are web-like structures formed by decondensed chromatins and granule proteins, serving to capture and eliminate pathogens in vitro and in vivo (Lee and Grinstein 2004; Brinkmann et al. 2004). However, Increasing evidence suggests that NETs exacerbate pathological processes in sterile inflammation, including atherosclerosis, thrombosis, and tumor metastasis (Kaplan and Radic 2012). It has been demonstrated that surgical stress induced by liver IR leads to the formation of NETs in the liver, further exacerbating liver injury in sterile inflammation. Whereas the peptidylarginine deaminase 4 (PAD4) inhibitors or DNase I has been shown to alleviate IRI in mice by inhibiting the formation of NETs (Huang et al. 2015).

Spleen tyrosine kinase (SYK), a non-receptor tyrosine kinase expressed in the cytoplasm, initially identified for its high expression in hematopoietic cells and recognized as a crucial target for the treatment of various autoimmune diseases (Mocsai et al. 2010; Liu and Mamorska-Dyga 2017). Recently, SYK has been shown to be expressed in non-hematopoietic tissues, including the liver, where it plays a central role in various hepatic signal transduction processes (Qu et al. 2018; Luci et al. 2022). For instance, our previous studies demonstrated that targeting SYK of monocyte-derived macrophages effectively alleviates the progression of liver fibrosis (Chen et al. 2021). However, the role of SYK in liver IRI remains unclear. GS-9973 is a SYK-specific small molecule inhibitor that has been shown to suppress fibrosis and HCC progression in a DEN-induced rat model of liver fibrosis (Qu et al. 2018). SYK is closely associated with the formation of NETs. Studies have shown that Asebogenin reduces neutrophil accumulation and NETs formation by interfering with the phosphorylation of SYK, thereby suppresses thrombus formation (Li et al. 2023a, b). P-selectin induces NETs through SYK/Ca^2+^/PAD4 signaling pathway to exacerbate acute pancreatitis (Xu et al. 2023). In a mouse CRLM model, activated neutrophils produce NETs that promote the occurrence of liver metastasis after surgical stress (Tohme et al. 2016).

Macrophages play an important role in maintaining liver homeostasis and have been identified as key regulators of liver inflammation. Monocyte-derived macrophages are plastic and diverse, and are participants and contributors to IR-associated inflammation. It has been shown that KCs are rapidly lost during liver injury, and a decrease in the number of KCs can be observed in metabolic dysfunction-associated steatohepatitis (MASH) and hepatocellular carcinoma models. At this time, monocyte-derived macrophages become the major contributor to replenish the liver macrophage pool and gradually begin to switch phenotypes (Rao et al. 2022; Wen et al. 2021). A studies have shown that selective targeting of SYK signaling in myeloid cells protects against liver fibrosis and hepatocarcinogenesis (Torres-Hernandez et al. 2019). One of our previous study showed that targeting SYK of monocyte-derived macrophages alleviates liver fibrosis by remodeling liver inflammatory environment (Chen et al. 2021).

We administered pharmacological inhibitors of SYK and specific targeting of mice liver macrophages to investigate the role of SYK in NETosis and the mechanism of its impact on liver injury and tumor recurrence in hepatic ischemia-reperfusion injury. In our study, we observed that excessive activation of SYK in neutrophils and macrophages during hepatic IRI promoted NETosis. GS-9973 reduced NETs formation by inhibiting the SYK/PKM2/P-STAT3 pathway in neutrophils and the NLRP3 inflammasome pathway dependent on IL-1β in macrophages, thereby alleviating hepatic IRI and liver cancer recurrence. In conclusion, targeting SYK may be a promising approach to mitigate hepatic ischemia-reperfusion injury and cancer recurrence.

## Methods

### Patient samples

Liver specimens from patients undergoing hepatectomy at Affiliated Yancheng School of Clinical Medicine of Nanjing Medical University were collected for this study. All patients donating samples for this project signed informed consents, and all experiments were approved by the Medical Ethics Committee of Yancheng Third People’s Hospital.

### Mouse liver partial warm ischemia models

6–8 weeks wild-type C57BL/6 male mice were fasted for at least 8 h before surgery and intraperitoneally injected with SYK inhibitors: GS-9973 (MCE, HY15968) (20 mg/kg), R406 (MCE, HY12067) (10 mg/kg), and Piceatannol (MCE, HY13518) (20 mg/kg) 4 h before surgery. The mice were induced with anesthesia using 2% isoflurane, followed by midline laparotomy. Heparin was injected, and non-traumatic vascular clamps were applied to occlude the arteries and portal veins of cephalic lobe, inducing 70% partial hepatic ischemia. After 90 min of partial hepatic ischemia, reperfusion was performed for 3 to 24 h. The sham-operated group underwent the same procedures without vascular occlusion.

### In vivo/in vitro SYK knockdown

In vivo: SYK siRNA (20 mg/kg, Santa Cruze, California, USA) was coupled with a mannose-conjugated polymer (polyplus transfection, Illkich, France), injected into the tail vein of mice 4 h before surgery and delivered to liver CD206hi macrophages.

In vitro: Bone marrow-derived macrophages were collected from C57BL/6 mice. The shSYK lentivirus (Genepharm, Shanghai, China) was synthesized to transfect BMDMs collected from C57BL/6 mice. 3 days after the induction of BMDMs, the cells were infected with 100 MOI shSYK-LV or NC-LV. After 24 h, stale medium were replaced by fresh medium with murine M-CSF (20 ng/mL). After 7 days, BMDMs were collected and used for subsequent experiments.

### Neutrophil isolation

Preoperative and postoperative peripheral blood was collected from patients or mice with hepatic IR and isolated with the EasySep Direct Human Neutrophil Isolation Kit (STEMCELL Technology, #19666) according to the manufacturer’s protocol. Mouse neutrophils were isolated using EasySep Mouse Neutrophil Enrichment Kit (STEMCELL Technology,#19762A). In some experiments, we treated neutrophils with SYK inhibitors (5µM).

### Isolation of neutrophils from Mouse liver

Preheated PBS was used to perfuse the liver (5 ml/min). After the liver became completely white, the liver was perfused with type IV collagenase (2 ml/min) dissolved in HBSS. After removing the gallbladder, the liver was harvested, and the digested cells were filtered with a 70 μm cell strainer to prepare a single-cell suspension. Cells were collected by centrifugation at 427 g for 10 min. 3 ml of 70% Percoll was added to the centrifuge tube, cells were resuspended on top of 8 ml of 40% Percoll added to 70% Percoll, and centrifuged at 872 g for 30 min. Cells on the 40%/70% Percoll boundary surface were washed twice for neutrophil separation.

### Extraction and cultivation of bone marrow-derived macrophages (BMDMs)

Bone marrow cells were isolated from the femurs and tibias of mice, filtered through a 70 μm cell strainer, followed by removal of erythrocytes with Red Blood Cell Lysis Buffer. Cells were cultured in DMEM medium supplemented with 10% FBS and 20% cell-free L929 medium. After one week, the medium was replaced for subsequent experiments.

### Flow cytometry

Isolated cells were preincubated with Fc receptor blockers to reduce nonspecific staining. Cells were fixed with Fixation/Permeabilization solution (BD Bioscience, 554722) at 4 ℃ for 20 min. After washing, cells were resuspended in BD Perm/Wash™ (BD Bioscience, 554723) buffer and incubated in the dark with the corresponding antibodies for 30 min: F4/80 (invitrogen, 2300247), CD11b (Biolegend, 101218), Ly6G (Biolegend, 127627), CD4 (Biolegend, 100443), FOXP3 (Biolegend, 126407). Flow cytometric analysis was performed using a flow cytometer (Beckman, California, CA, USA), and the data were analyzed using FlowJo (version 10.8.1).

### Co-immunoprecipitation

The isolated peripheral blood neutrophils were lysed with NP40 for 30 min, centrifuged at 12,000 g for 15 min to collect the supernatant. The cell lysates were immunoprecipitated using primary antibodies and Protein A + G Agarose. Total lysates or immunoprecipitated complexes were subsequently subjected to SDS-PAGE electrophoresis, followed by immunoblot using corresponding antibodies.

### Western blot

Liver tissues or cells were lysed using RIPA, separated by SDS-PAGE gel electrophoresis and then transferred to PVDF membranes. The proteins were then immunoblotted with corresponding primary antibody: anti-P-SYK (Tyr525/526) (Cell Signaling Technology, 2710), anti-SYK (Cell Signaling Technology, 2712), anti-PKM2 (Cell Signaling Technology, 4053), anti-P-STAT3 (Cell Signaling Technology, 9145), anti-β-actin (Cell Signaling Technology, 4970), anti-MPO (ab208670, Abcam), anti-CitH3 (ab281584, Abcam), anti-NE (bs-6982R, Bioss), anti-β-actin (Cell Signaling Technology, 4970), using chemiluminescence-based method for color development.

### qPCR

RNA was extracted from tissues or cells and reverse transcribed into cDNA. Real-time fluorescence quantitative polymerase chain reaction was conduct using SYBR green master mix. all expression levels of target genes were normalized to β-actin expression.

### Determination of serum ALT, AST

The blood samples collected from patients or mice were stored at 4 °C overnight and then centrifuged at 300 rpm for 15 min. The levels of alanine aminotransferase (ALT) and aspartate aminotransferase (AST) levels in the serum were determined using corresponding kits (Servicebio, GM1102, GM1103). The results were measured using an automatic biochemical analyzer (Rayto Life and Analytical Sciences Co., Ltd., Chemray 240).

### Histopathology

The liver tissue specimens were fixed in 4% formaldehyde, embedded in paraffin, and sectioned into 4 μm thick slices for hematoxylin-eosin (H&E) staining. The severity of liver injury was graded based on the Suzuki scoring system.

### Immunohistochemistry and Immunofluorescence

For Immunofluorescence, the fixed tissue sections were placed in a repair kit (Servicebio, China) filled with EDTA antigen retrieval buffer (PH = 9.0), and then boiled in a microwave for repair. BSA (3%) solution was added for 30 min to block nonspecific binding, and then the corresponding primary antibodies were added: anti-P-SYK (Bioss, bs-3434R), anti-PKM2 (Cell Signaling Technology, 4053), anti-P-STAT3 (Cell Signaling Technology, 9145), anti-MPO (Abcam, ab208670), anti-CitH3 (Abcam, ab281584).

### TUNEL assay

Freshly frozen liver tissues were collected and stained using a TUNEL kit (Servicebio, G1504-50T), and images were collected using a fluorescence microscope to calculate the rate of positively stained cells.

### ELISA assay

Murine serum and cell culture supernatant was collected, and the levels of the cytokine were measured using a mouse IL-1β ELISA kit (Abcam, ab197742).

### Statistical analysis

The data are expressed as mean ± SD, and the statistical difference between subgroups is determined by GraphPad Prism. Statistical significance was analyzed using the Student’s unpaired t test. Linear regression is used to assess the strength of the linear relationship between variables. Two-tailed *P*-value less than 0.05 is considered statistically significant.

## Results

### Drug screening identified GS-9973 as the most potent SYK inhibitor for alleviating hepatic IRI

**Fig. 1:**
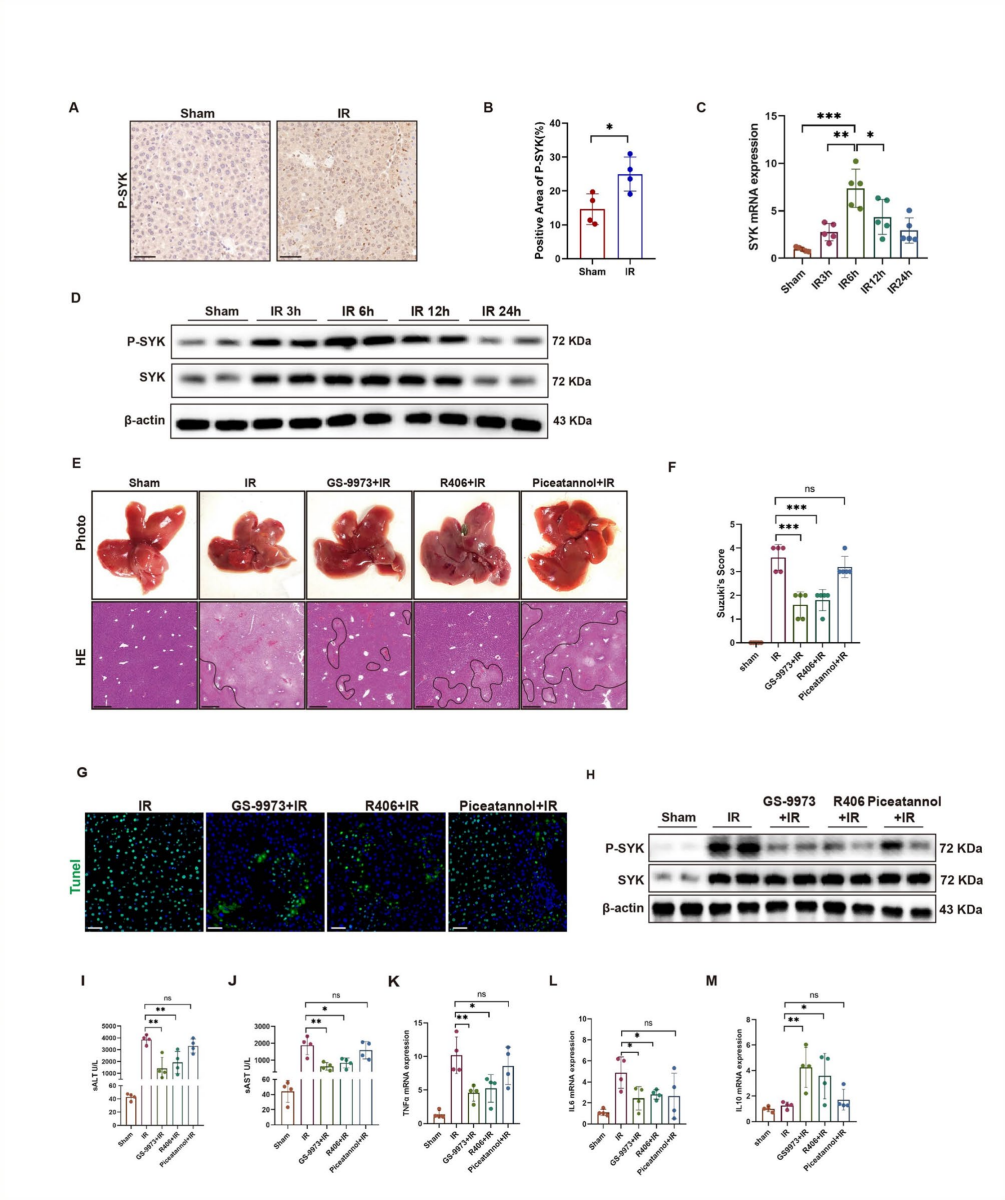
SYK inhibitor alleviates hepatic IR injury in mice. 6–8 weeks mice were intraperitoneally injected with SYK inhibitor before 70% hepatic ischemia, followed by reperfusion for 3, 6, 12 and 24 h. **A** Immunohistochemistry staining of P-SYK in 6 h IR liver tissues or sham liver tissues, scale bars, 50 μm (*n* = 4). **B** Quantification of hepatic P-SYK immunohistochemistry (*n* = 4). **C** The expression of SYK in the liver at different time points after IR was detected by qPCR (*n* = 5). **D** Western Blot was used to analyze the protein level of P-SYK and SYK in livers (*n* = 4). **E** Representative HE staining of IR liver tissue. Scale bars, 50 μm (*n* = 4). **F** Suzuki histologic criteria were used to assess liver tissue injury induced by IR (*n* = 5). **G** Apoptosis of liver cells was detected by tunel staining. Scale bars, 20 μm (*n* = 4). **H** The expression of P-SYK in liver tissue was detected by Western Blot (*n* = 4). **I**,** J** Serum ALT and AST levels in mice (*n* = 4). **K-M** The mRNA levels of TNFα, IL6, IL10 were detected by qPCR (*n* = 4). ∗*P* < 0.05; ∗∗*P* < 0.01; ∗∗∗*P* < 0.001

Based on our previous research, blocking SYK in monocyte-derived macrophages reduces the production of CXCL1 and M2 polarization of macrophages, thus alleviating chronic inflammation and fibrosis in the liver. Moreover, Trem2 in monocyte-derived macrophages promotes IR inflammatory regression by regulating Cox2/PGE2/Rac1 pathway mediated efferocytosis, and blocking myeloid Trem2 alleviates early liver injury induced by IR (Han et al. 2023). Trem2 activates SYK phosphorylation via Dap12 to induce the inflammatory cascade in macrophages is known to be the classical paradigm. Upon receiving an extracellular stimulus, Trem2 associates with the adapter DAP12, which recruits and activates SYK through its cytoplasmic immunoreceptor tyrosine-based activation motif (ITAM) (Wang and Colonna 2023; Wang et al. 2022). Therefore, we were curious about the role of SYK in hepatic IR. We observed a significant upregulation of P-SYK and SYK expression in the livers of mice undergoing IR (Fig. 1A-D). Furthermore, the most severe liver damage was observed 6 h after liver reperfusion, coinciding with a significant increase in P-SYK expression (Fig. 1D, Figure S1A-C). In this study, we evaluated the effects of three SYK inhibitors on hepatic IRI: GS-9973, R406, and Piceatannol, which have now been shown to have therapeutic promise in hematologic tumors and autoimmune diseases (Liu and Mamorska-Dyga 2017; Currie et al. 2014; Suljagic et al. 2010). Administration of SYK inhibitors in mice prior to IR 6 h significantly inhibited SYK phosphorylation and alleviated liver tissue damage to different degrees (Fig. 1E-H). Among them, mice injected intraperitoneally with GS-9973 and R406 showed less liver tissue damage and a decrease in serum liver enzymes levels than mice treated with Piceatannol, and GS-9973 exhibited the most effective inhibitory effect (Fig. 1I, J). At the same time, the mRNA levels of proinflammatory factors TNFα and IL6 were down-regulated (Fig. 1K, L), and the transcription of IL10 was increased in liver tissue (Fig. 1M).

### GS-9973 most effectively inhibits the formation of IR-related NETs

**Fig. 2:**
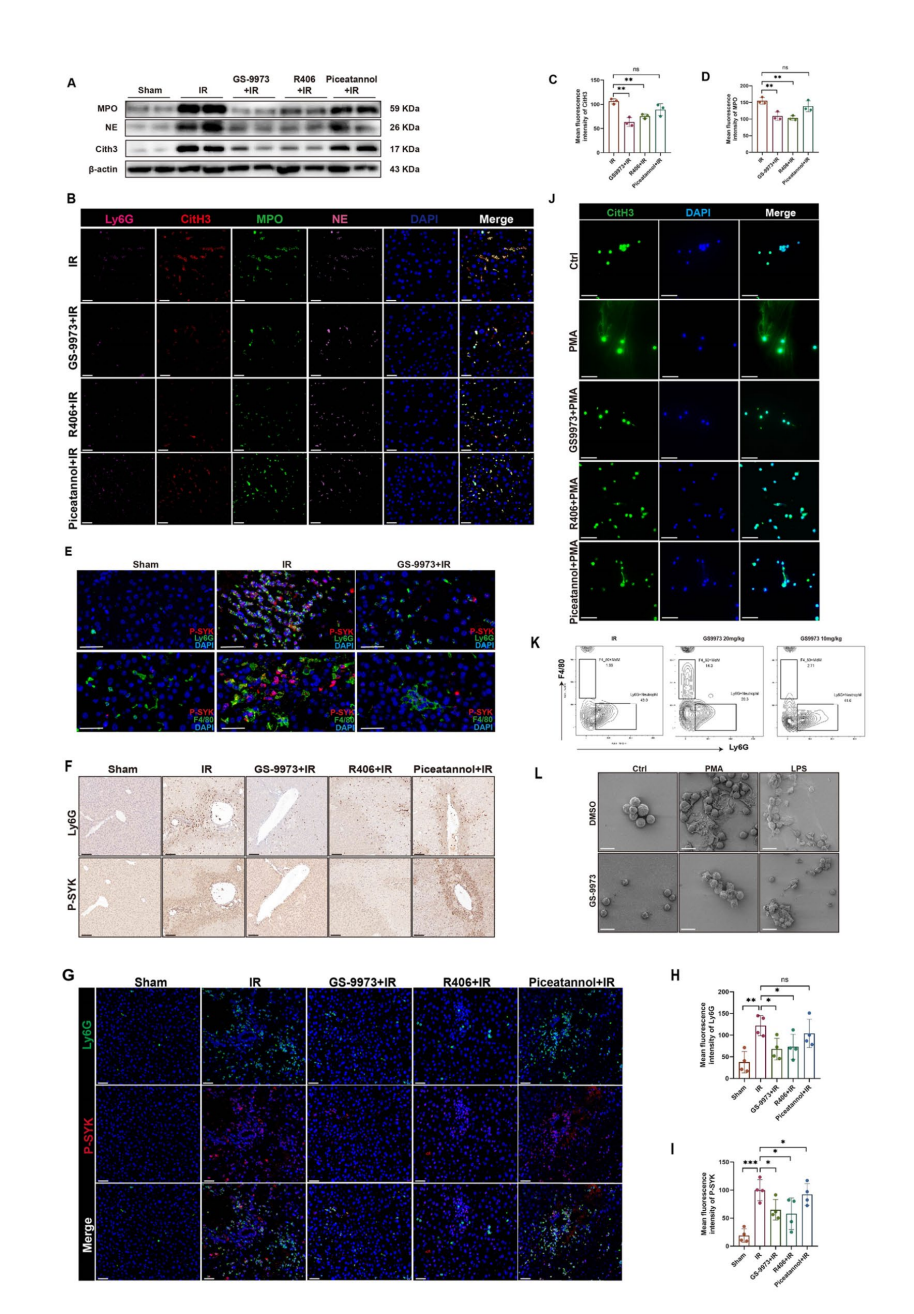
Inhibition of SYK reduces neutrophil recruitment and NETs formation during hepatic IR. Mice were injected with three different SYK inhibitors prior to hepatic portal occlusion, followed by 6 h of reperfusion before harvesting mouse liver tissues. **A**,** B** The expression levels of MPO, NE and CitH3 were detected in mouse liver tissues by Western Blot and immunofluorescence. Scale bars, 20 μm (*n* = 4). **C**,** D** Quantification of fluorescence intensity of CitH3 and MPO by Image J (*n* = 3).**E** Immunofluorescence was performed to detect the co-localization of P-SYK and Ly6G, as well as P-SYK and F4/80 in mouse liver tissues. Scale bars,40 μm (*n* = 4). **F** Immunohistochemistry was conducted to assess the expression of Ly6G and P-SYK. Scale bars,100 μm (*n* = 3). **G** Confocal immunofluorescence staining of P-SYK and Ly6G were performed in livers. Scale bars,40 μm (*n* = 4). **H**,** I** Quantification of fluorescence intensity of Ly6G and P-SYK by Image J (*n* = 4). **J** Neutrophils were isolated from mouse bone marrow and pretreated with the SYK inhibitors (5µM) for 2 h before stimulation with 100nM PMA for 3 h. The expression of CitH3 was observed using fluorescence confocal microscopy. Scale bars,50 μm (*n* = 4). **K** The mice were intraperitoneally injected with 20 mg/kg or 10 mg/kg GS-9973 4 h before liver ischemia, followed by 6 h of reperfusion. Subsequently, liver perfusion was conducted, and non-parenchymal cells were isolated from livers for flow cytometry analysis to detect the proportion of neutrophils in the liver (*n* = 4). **L** Neutrophils pretreated with GS9973 were stimulated with 100nM PMA or 100ng/ml LPS, and NETs formation was observed by scanning electron microscopy, with DMSO as control. Scale bars,10 μm (*n* = 4). ∗*P* < 0.05; ∗∗*P* < 0.01; ∗∗∗*P* < 0.001

NETs play a crucial role in regulating the inflammatory response in hepatic IRI. Studies have indicated that SYK phosphorylation at Tyr525/526 is important for the production and function of NETs (Li et al. 2023a, b). We collected neutrophils in liver tissue from mice 6 h after liver surgery and found increased formation of NETs compared to normal liver tissue (Figure S2A, B). We found that GS-9973 was the most effective in reducing the formation of IR-related NETs in mice compared to the other two SYK inhibitors (Fig. 2A-D). Subsequently, we wondered whether GS-9973 targets and acts on P-SYK localized in neutrophils. Our research indicates that P-SYK is predominantly upregulated in neutrophils in response to liver IR in mice, and this upregulation is significantly inhibited by GS-9973 (Fig. 2E). Expression of P-SYK is also increased in macrophages (Fig. 2E), while no significant expression was observed in hepatocytes (Figure S2C). Furthermore, GS-9973 simultaneously reduces the expression of CXCL1/CXCL2 (Figure S1D, E). We therefore wondered whether pharmacological inhibition of SYK suppresses NETs production by affecting neutrophil recruitment. Thus, we intraperitoneally injected SYK inhibitors into mice prior to liver IR, and showed that inhibition of SYK significantly reduced the number of neutrophils in the liver (Fig. 2F, G, H, I,Figure S1F), and the concentration of in vivo dosing affected its recruitment efficacy (Fig. 2K). The level of P-SYK was positively correlated with the level of Ly6G (Figure S1G). We next wondered whether GS-9973 regulates the pathologic process of NETs formation. We pretreated mouse bone marrow neutrophils with three SYK inhibitors in vitro for 2 h, followed by stimulation with PMA and LPS. We found a reduction in NETs production compared to the control group, with GS-9973 showing a higher inhibitory effect on NETs (Fig. 2J, L). GS-9973 clearly demonstrated inhibition of SYK activation in vitro (Figure S2D, E). Consistent results were also obtained in human peripheral blood neutrophils (Figure S2F). Therefore, we demonstrate that GS-9973 not only inhibits neutrophil recruitment but also directly acts on neutrophils to regulate NETs formation.

### The formation of NETs regulated by SYK depends on PKM2/P-STAT3

**Fig. 3:**
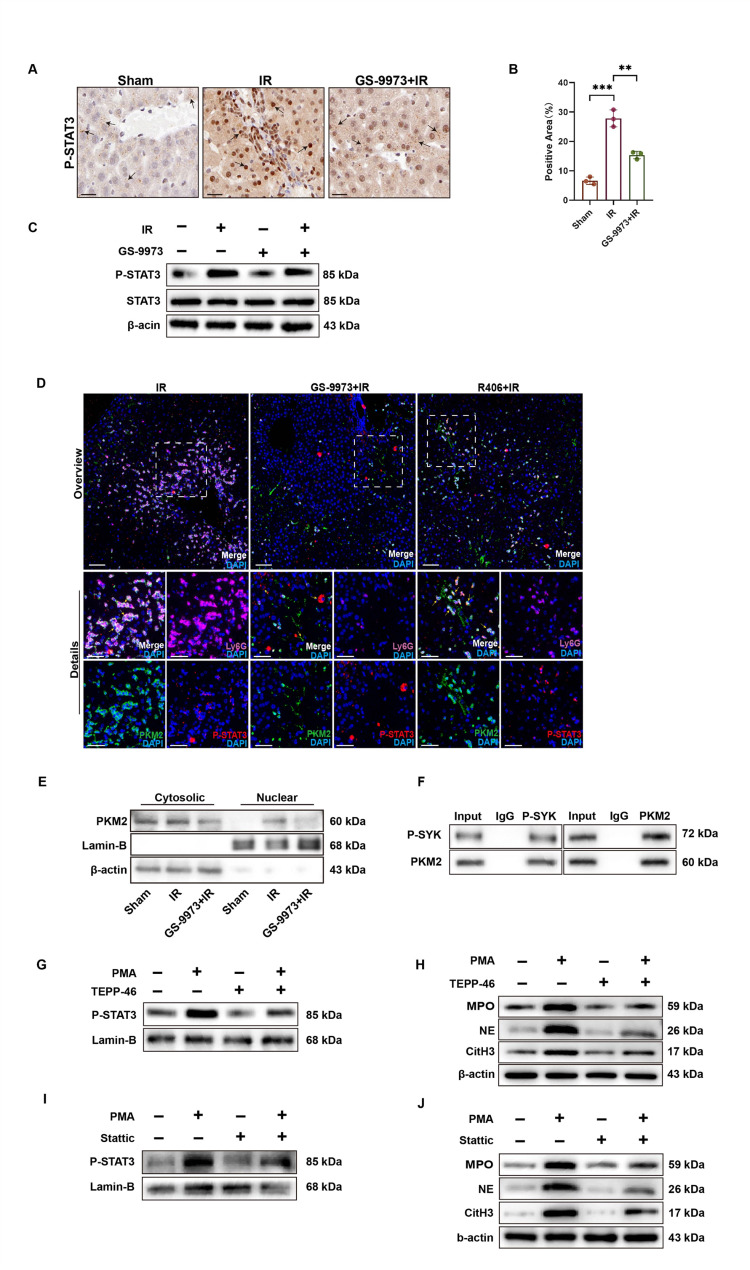
SYK regulates the formation of NETs through the PKM2/P-STAT3 pathway. Mice were intraperitoneally injected with GS-9973 4 h before liver IR. **A** Immunohistochemical analysis was conducted on postoperative mouse liver tissues to assess the expression of P-STAT3 and STAT3. Scale bars, 20 μm (*n* = 3). **B** Quantitative analysis of immunohistochemical positive areas of P-STAT3 (*n* = 3). **C** The expression of P-STAT3 in liver tissue analysed by Western Blot (*n* = 4). **D** Immunofluorescence staining was performed to detect the expression of PKM2, P-STAT3, and Ly6G in liver tissues. Scale bars, 40 μm (*n* = 4). **E** Neutrophils were isolated from the blood of mice at 6 h after IR, and the expression of PKM2 in the cytoplasm and nuclear proteins was detected by Western Blot (*n* = 4). **F** Immunoprecipitation of neutrophil lysates from the peripheral blood of patients after partial hepatectomy was performed with anti-P-SYK antibody, and the interaction between P-SYK and PKM2 in the Co-IP complex was detected by Western Blot (*n* = 4). **G** Neutrophils were treated with PMA and TEPP-46, and the expression of P-STAT3 in the nucleus was detected by Western Blot (*n* = 4). **H** Western Blot was used to detect the expression of MPO, NE, and CitH3 in neutrophils (*n* = 4). **I** Neutrophils were treated with PMA and Stattic, and the expression of P-STAT3 in the nucleus was detected by Western Blot (*n* = 4). **J** Western Blot was used to detect the expression of MPO, NE, and CitH3 in neutrophils (*n* = 4). ∗∗*P* < 0.01; ∗∗∗*P* < 0.001

STAT3 is a key participant in NETosis induced by liver inflammation, as experiments have shown that STAT3 overexpression or PMA treatment both promote the formation of NETs in the colon cancer-derived neutrophils (Zhang et al. 2023). PKM2 mainly exists in the cytoplasm in the form of a tetramer, which plays a pyruvate kinase activity. When receiving stimulus signals, PKM2 is allosteric transferred to the nucleus in the form of a dimer, which plays a protein kinase activity and regulates the transcription and expression of downstream genes, including proinflammatory genes. PKM2 entering the nucleus can activate STAT3 and promote the inflammatory process (Palsson-McDermott et al. 2015; Gao et al. 2022). We found that nuclear translocation and expression of P-STAT3 in the liver after IR were significantly increased and markedly inhibited by GS-9973 (Fig. 3A-C). We then inquired whether the inhibitory effect of GS-9973 on P-STAT3 depends on the inhibition of PKM2 nuclear translocation. It is known that SYK-dependent phosphorylation of PKM2 at Tyr105 and its nuclear translocation regulate CRC-associated MDSCs (Zhang et al. 2022). The expression of PKM2 was significantly up-regulated in neutrophils in response to IRI, and its nuclear translocation was increased. GS-9973 treatment reduced the nuclear expression of PKM2 and inhibited the nuclear translocation of P-STAT3 (Figure S2H). In addition, confocal immunofluorescence showed that PKM2 and P-STAT3 were co-localized in the nucleus of neutrophils in response to liver inflammation, while GS-9973 inhibited the interaction between PKM2 and P-STAT3 in the nucleus (Fig. 3D-E). To further confirm the interaction between SYK and PKM2, we performed co-immunoprecipitation of neutrophils from patients undergoing hepatic portal occlusion and demonstrated that the stimulation of IRI promoted the interaction of P-SYK with PKM2 (Fig. 3F). TEPP-46, as a known allosteric activator of PKM2, can lead to its tetramerization, block its nuclear translocation, and reduce STAT3 phosphorylation by inhibiting nuclear PKM2 accumulation (Angiari et al. 2020; Shen et al. 2023). We observed that neutrophils treated with TEPP-46 detected lower levels of P-STAT3 and NETs production in response to PMA stimulation relative to controls (Fig. 3G, H). Stattic is a potent STAT3 inhibitor known to suppress STAT3 phosphorylation and activation. Treatment with Stattic resulted in a decrease in the production of P-STAT3 and NETs in neutrophils stimulated with PMA (Fig. 3I, J). In vivo experiments also showed that preoperative administration of TEPP-46 and Stattic had a protective effect on liver IRI in mice (Figure S3D, E). Additionally, the combined use of TEPP-46 and Stattic not further significantly reduce NETs formation (Figure S3A-C). Therefore, we conclude that GS-9973 regulates NETs production through the PKM2/P-STAT3 pathway, thereby affecting hepatic IRI.

### Targeting SYK of monocyte-derived macrophages reduces neutrophil recruitment and IL1β-mediated NETosis

**Fig. 4:**
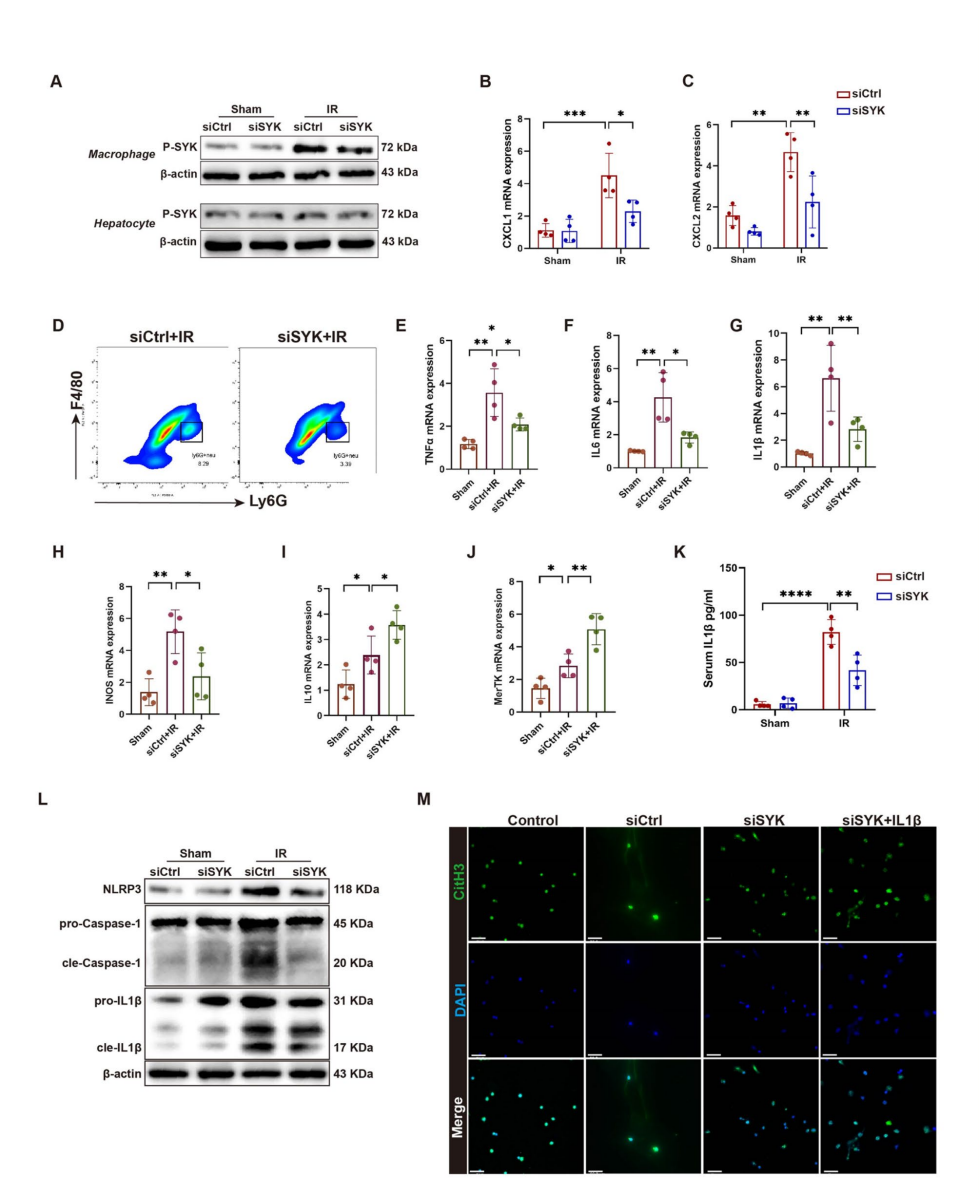
Targeting SYK of monocyte-derived macrophages reduces NETs formation. Mice were injected with a mixture of siSYK and mannose-conjugated polymer before IR, and liver tissues were extracted 6 h after reperfusion. **A** The P-SYK expression was detected by western blot in primary hepatocytes or macrophages isolated from ischemia livers or sham livers (*n* = 4). **B**,** C** Detection of CXCL1 and CXCL2 mRNA levels in liver tissue by qPCR (*n* = 4). **D** After liver perfusion, hepatic nonparenchymal cells were isolated, and the number of Ly6G^+^ cells was detected by flow cytometry (*n* = 4). **E-J** Detection of transcript levels of TNFα, IL6, IL1β, INOS, IL10 and MerTK in liver tissue by qPCR (*n* = 4). **K** ELISA was used to measure the serum levels of IL1β after IR in siCtrl group and siSYK group (*n* = 4). **L** Western Blot was used to detect the protein levels of NLRP3, IL1β and Caspase-1 in the liver tissue of mice (*n* = 4). **M** BMDMs were transfected with shSYK in advance, and the transfected BMDMs were treated with LPS + ATP, then switched to fresh medium without LPS or ATP for culture. After 1 h, neutrophils were transferred to macrophage medium, and NETs formation was detected by immunofluorescence after 3 h. Scale bars, 50 μm (*n* = 4). ∗*P* < 0.05; ∗∗*P* < 0.01; ∗∗∗*P* < 0.001;****P < 0.0001

The expression of P-SYK was also upregulated in macrophages in IR livers and was inhibited by GS-9973 (Fig. 2E), prompting our interest in understanding the role and mechanism of macrophages in this process. GS-9973 has previously been shown to reduce the recruitment of neutrophils in liver IRI, We wondered about whether GS-9973 influences neutrophil recruitment by regulating SYK in macrophages. We injected siSYK mixed with mannose-conjugated polymer (JetPEI-polyplus) in mice via tail vein before IR to knockdown SYK in liver monocyte-derived macrophages(MoMFs), This method was elaborated in our previous study (Chen et al. 2021; Wang et al. 2021a, b). The efficiency of knockdown targeting macrophages rather than hepatocytes in vivo was verified by Western Blot (Fig. 4A). We found that targeting of MoMF SYK also reduced the transcription of CXCL1 and CXCL2 and neutrophil recruitment in the liver after IR (Fig. 4B-D). The expression of S100A9 in liver was also down-regulated by siSYK (Figure S2G). Moreover, we found that the levels of proinflammatory cytokines TNFα, IL6, IL1β and INOS, especially IL1β, were significantly down-regulated in the liver (Fig. 4E-H), and the level of IL10 and MerTK were up-regulated (Fig. 4I, J). The level of IL1β in the serum of mice was also significantly down-regulated (Fig. 4K). IL1β is mainly activated by the NLRP3 inflammasome, and studies have shown that macrophage-derived NLRP3 inflammasome activation and IL1β release can induce NETosis (Yalcinkaya et al. 2023). SYK has been shown to activate the NLRP3 inflammasome in many studies. Here, we hypothesized that macrophage SYK may regulate NLRP3 inflammasome activation, affecting the production of IL1β, and thereby regulating NETosis. We found that inhibition of SYK in macrophages significantly suppressed the expression of NLRP3, cleaved Caspase-1, cleaved IL1β in mouse liver (Fig. 4L). Knockdown of SYK in raw264.7 cells resulted in decreased secretion of IL1β in response to LPS in vitro (Figure S3F). Pre-treatment of mice with MCC950 (a NLRP3 inflammasome inhibitor) (20 mg/kg, ip) or Anakinra (an interleukin-1 receptor (IL-1R) antagonist) (20 mg/kg, ip) before IR, alleviated liver injury after IR (Figure S3G, H), reduced the production of NLRP3 inflammasomes (Figure S3I), and diminished NETs formation in the liver (Figure S3J-L). In vitro, BMDMs transfected with shSYK were treated with LPS + ATP, followed by incubation in fresh medium without LPS or ATP for 1 h to allow the release of pro-inflammatory factors. Then, neutrophils were transferred to the macrophage culture medium. We observed that knocking down SYK in macrophages reduced the NETs formation induced by macrophages. Addition of IL1β (100 ng/ml) (Sigma, I5271-5UG) reversed the inhibitory effect of GS-9973 on NETs formation (Fig. 4M). These results suggest that GS-9973 can diminish NETs formation by inhibiting SYK-mediated activation of the NLRP3 inflammasome and IL1β in macrophages.

### GS-9973 alleviates IR-driven liver tumor recurrence

**Fig. 5:**
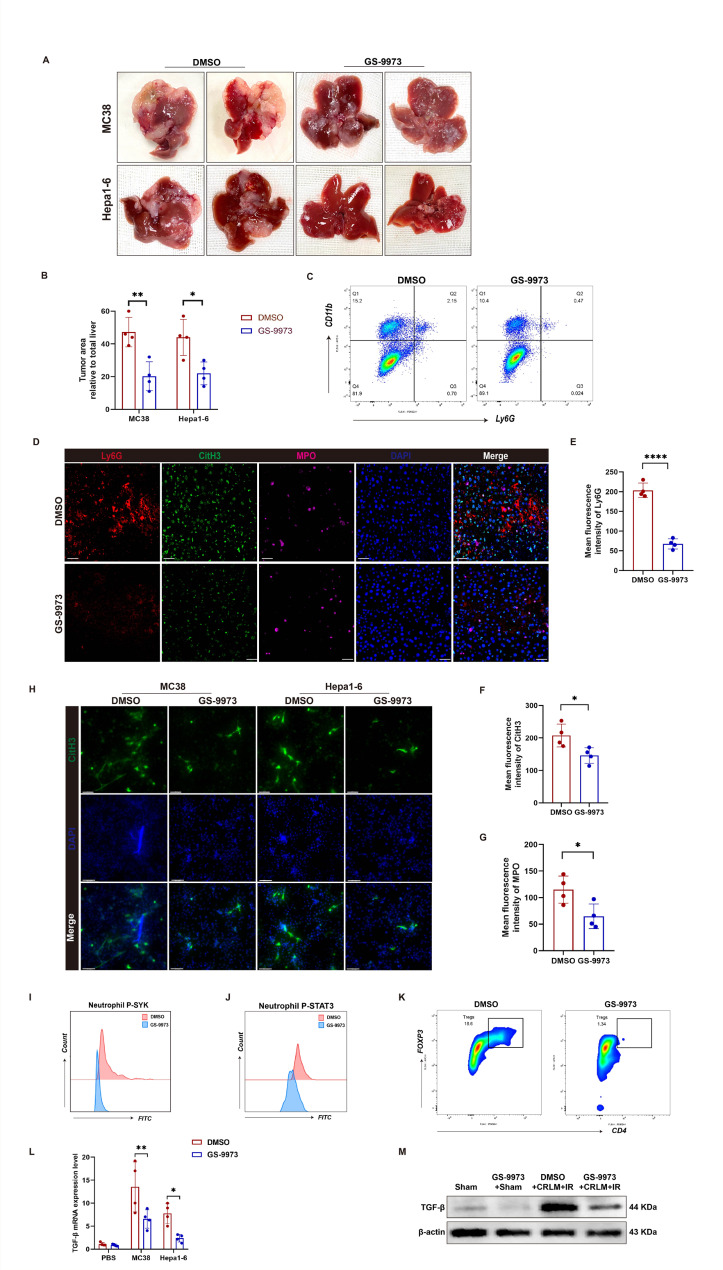
GS-9973 reduces liver tumor recurrence by reducing NETs formation and Treg cell differentiation. Mice were injected with GS-9973 or DMSO 4 h before spleen injection of MC38 or Hepa1-6 cells, followed by CRLM or HCC recurrence and 70% IR surgery. After administering GS9973 once every three days for two weeks, the mice were sacrificed and the liver tumors were assessed. **A** Liver tumor metastasis or recurrence of mice in control group and GS-9973 injection group (*n* = 4). **B** Tumor area (*n* = 4). **C** Mouse liver tumor cells were extracted and the proportion of Ly6G^+^CD11b^+^ neutrophils was detected by flow cytometry (*n* = 4). **D-G** The expressions of Ly6G, CitH3 and MPO in liver tissue were detected by immunofluorescence and quantified by image J. Scale bars, 20 μm (*n* = 4). **H** Murine bone marrow neutrophils were pretreated with GS-9973 (5µM) or DMSO for 2 h and co-cultured with MC38 cells or Hepa1-6 cells at a ratio of 1:10 for 4 h. The expression of Cith3 was detected by immunofluorescence microscopy. Scale bars, 50 μm (*n* = 4). **I**,** J** The proportion of P-SYK^+^ or P-STAT3^+^ cells was gated in neutrophils by flow cytometry (*n* = 4). **K** Flow cytometry was employed to detect the proportion of CD4^+^FOXP3^+^ Treg cells in tumor tissue (*n* = 4). **L** The mRNA levels of TGFβ in liver tissue was detected by qPCR (*n* = 4). **M** The protein of TGFβ in liver tissue was detected by Western Blot (*n* = 4). ∗*P* < 0.05; ∗∗*P* < 0.01; ****P < 0.0001

We have previously shown that targeting SGK1 alleviates recurrence of liver metastasis after hepatic IR (Li et al. 2023a, b). To investigate the effect of pharmacological inhibition of SYK on liver tumor recurrence after IRI, we established standardized mouse models of colorectal liver metastasis (CRLM) and HCC recurrence. Mice were pre-treated with GS-9973 via intraperitoneal injection, followed by splenic injection of MC38 or Hepa1-6 cells 4 h later. After 15 min of circulation, the spleen was ligated and removed, and 70% hepatic ischemia-reperfusion was performed simultaneously. GS-9973 was intraperitoneally injected once 3 days after surgery, and the liver was harvested 2 weeks later. We observed that GS-9973 significantly reduced liver tumor metastasis and recurrence (Fig. 5A, B), and detected a decrease in NETs formation in liver tissues (Fig. 5D-G), a reduction in the proportion of neutrophils in tumor tissues (Fig. 5C). Furthermore, the phosphorylation levels of SYK and STAT3 in neutrophils were notably downregulated (Fig. 5I, J). Mouse bone marrow neutrophils were pretreated with GS-9973 or DMSO and co-cultured with MC38 or Hepa1-6 cells. The results showed that GS-9973 significantly reduced the formation of NETs in the co-culture system (Fig. 5H). It is known that Tumor-associated neutrophils(TAN) in HCC induce CD4^+^ T cells to express TGFβ, which promotes Treg cell differentiation and T cell exhaustion, thereby suppressing anti-tumor immune responses (Geh et al. 2022). We also observed a decrease in the number of Treg cells in tumor tissues after inhibiting SYK (Fig. 5K). Meanwhile, the mRNA and protein levels of TGFβ were significantly down-regulated in liver tumor tissues of GS9973-treated mice (Fig. 5L, M). Therefore, we speculate that inhibiting SYK mitigates liver tumor metastasis by reducing the recruitment of neutrophils and the formation of NETs. Additionally, by downregulating TGFβ, it affects the recruitment of Treg cells in tumor tissue, further reducing tumor metastasis.

### Expression of peripheral P-SYK associated with the prognosis of patients after liver surgery

**Fig. 6:**
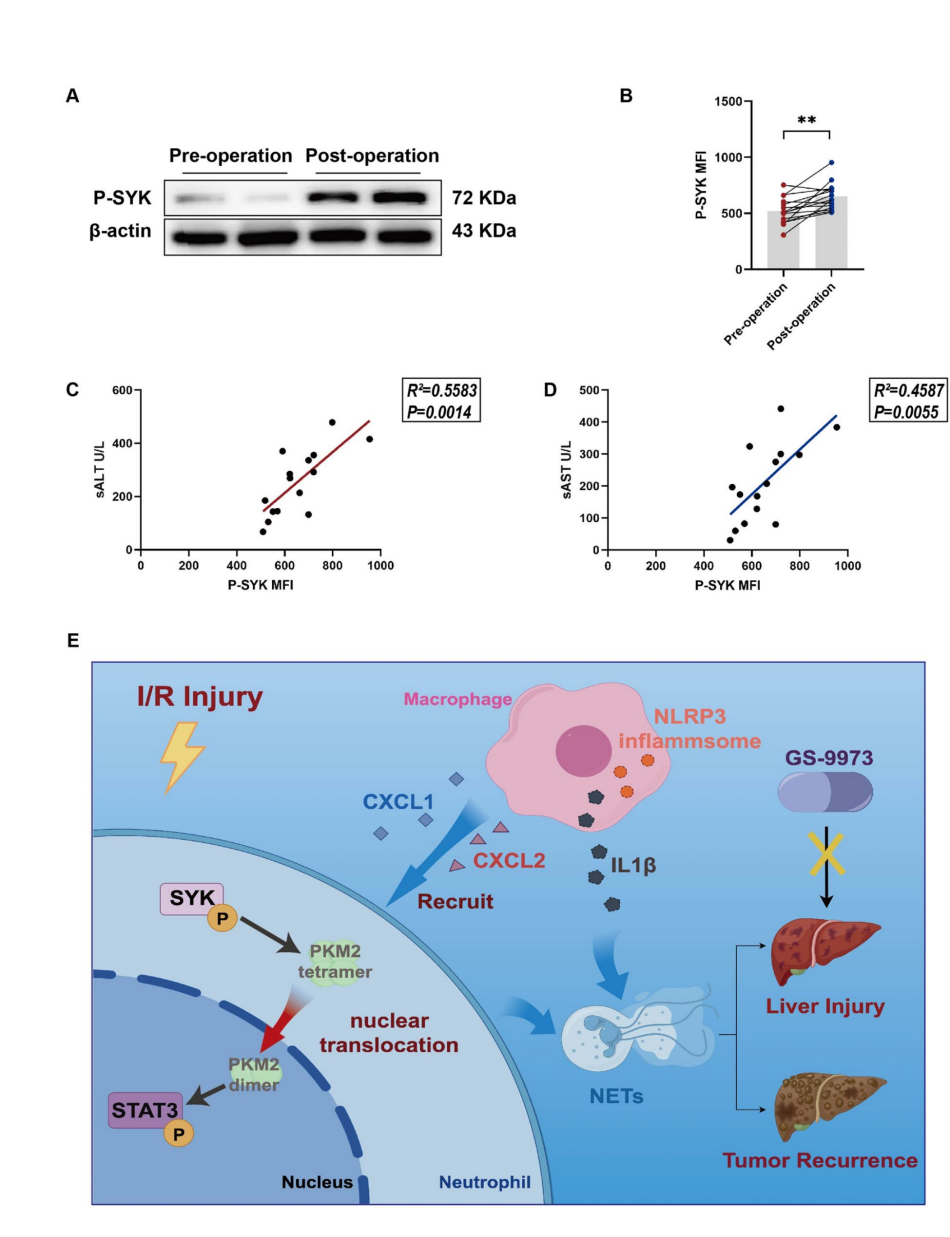
The expression of peripheral SYK predicts the prognosis of patients after liver resection. Neutrophils were extracted from the peripheral blood of 15 patients 1 day before and 1 day after partial hepatectomyand. **A** The expression of P-SYK in neutrophils was detected by Western Blot (*n* = 4). **B** Flow cytometry was used to measure the MFI of P-SYK in neutrophils (*n* = 15). **C**,** D** The levels of serum ALT and AST were detected and their correlation with the MFI of P-SYK was analyzed (*n* = 15). **E** Schematic diagram of the signaling pathway of SYK in hepatic IR. P-SYK is up-regulated in neutrophils and macrophages in response to hepatic IR. Neutrophil SYK regulates NETs formation through PKM2/P-STAT3. Macrophage SYK promotes NETs formation by promoting neutrophil recruitment and regulating NLRP3-dependent IL1β pathway. GS-9973 targeted SYK to alleviate hepatic IR and liver tumor recurrence by blocking the above pathways. ∗∗*P* < 0.01

To explore whether the abundance of P-SYK can serve as a predictor for the prognosis of patients undergoing partial hepatectomy, we collected peripheral blood samples from patients on the first day after hepatectomy, and measured the mean fluorescence intensity (MFI) of P-SYK in peripheral blood neutrophils by flow cytometry. We found that P-SYK expression in neutrophils was significantly increased postoperatively compared to preoperative levels (Fig. 6A-B). Moreover, the MFI of P-SYK in peripheral blood was positively correlated with the postoperative serum ALT and AST levels (Fig. 6C-D). In summary, our study suggests that SYK in neutrophils and macrophages is a promising target for alleviating liver IR injury and preventing liver tumor recurrence. GS-9973, as a specific small molecule inhibitor of SYK, may provide additional benefits for patients with liver cancer (Fig. 6E).

## Disscussion

Hepatic ischemia reperfusion injury is an important factor inducing liver dysfunction and reduced survival rate in patients after liver surgery. During hepatic IRI, innate immune cells including monocytes and neutrophils are recruited to the damaged liver as pioneers in response to chemokines produced by the damaged tissue to synergistically modulate the inflammatory cascade, induce hepatocyte apoptosis or further orchestrating tissue repair and organ damage. Neutrophils are the largest population of immune cells in the human body and are the first to arrive at the battlefield during aseptic liver injury. Numerous studies have shown that, in addition to the classical role of neutrophils, neutrophil-derived NETs play an important role in the pathological process of hepatic IR (Huang et al. 2015); Kaltenmeier et al. 2022). NETs are released by neutrophils and are a unique function of neutrophils to capture or defend against pathogens. In this study, we detected high P-SYK expression and dysregulated NETs formation in both humans and mice undergoing liver IR, so we focused on the relationship between overactivated SYK in neutrophils and NETosis in this study.

First, we demonstrated that the Syk-specific small molecule inhibitor GS-9973 exerts an remarkable inhibitory effect on IR-induced NETs activation both in vivo and in cell culture studies. Pharmacological inhibition of SYK significantly alleviated NETs formation in the liver of mice with IRI. This is consistent with the conclusion that Asebogenin targets SYK to reduce NETs formation in thrombosis (Li et al. 2023a, b). PKM2 in neutrophils has been shown to be upregulated and promote NETs activation during cerebral ischemia-reperfusion (Dhanesha et al. 2022). PKM2 exists as a tetramer and dimer, and it is present as a tetramer in the cytoplasm, where it exerts pyruvate kinase activity. Upon extracellular stimulation, PKM2 translocates to the nucleus as a dimer, demonstrating protein kinase activity, and can phosphorylate STAT3 directly, independent of the JAK2 and C-Src pathways, regulating downstream gene transcription and expression (Hou et al. 2020; Gao et al. 2012). Administration of SYK inhibitor before IR in mice reduced activation of PKM2 and P-STAT3 and downregulation of their nuclear expression in the injured liver. Nuclear PKM2 has been demonstrated to bind P-STAT3 and promote STAT3 phosphorylation, thereby regulating the expression of inflammatory genes and the production of pro-inflammatory cytokines in a variety of cells (Palsson-McDermott et al. 2015; Gao et al. 2022). STAT3 is a crucial participant in hepatic inflammation, and the role of activated STAT3 in hepatic IRI has been extensively studied (Yang et al. 2023). In a mouse model of hepatic IRI, pharmacologic inhibition of PKM2 nuclear translocation by TEPP-46 resulted in inhibition of STAT3 activation in neutrophils. These evidences suggest that SYK regulates NETs formation via the neutrophil PKM2/P-STAT3 pathway.

Additionally, we observed upregulation of P-SYK in macrophages of the reperfused liver. Therefore, we attempted to explore whether the upregulation of P-SYK in macrophages contributes to the inflammation in IRI. The SYK siRNA was coupled with a mannose-conjugated polymer, and injected into the tail vein of mice to Delivery to liver Monocyte-derived macrophages. The effect of intervening in monocyte-derived macrophages using mannose-conjugated polymer carrying siSYK has been extensively studied in hepatic IRI (Chen et al. 2021; Wang et al. 2021a, b). We found that specific targeting of macrophage SYK also resulted in significant remission of hepatic IRI. Silencing SYK in macrophages downregulates CXCL1/CXCL2 and reduces NLRP3 inflammasome activation and IL1β production. IL1β is a SYK-dependent cytokine. The production of mature IL1β requires NFκB-mediated transcription of pro-IL1β and proteolytic processing of pro-IL1β by activated caspase-1, which is an important signal to promote NETosis (Mocsai et al. 2010). Moreover, the synthesis of pro-IL-1β is regulated by the SYK-CARD9 pathway, while NLRP3 activation depends largely on SYK rather than CARD9. More and more studies have shown that the activation of NLRP3 and IL1β by SYK is reflected in multiple disease models (Shio et al. 2009). For example, SYK has been shown to control pro-IL1β synthesis and NLRP3 activation in fungal infections and Alzheimer’s disease (Gross et al. 2009; Jung et al. 2022). However, SYK may have a more extensive function in macrophages, but at least in part by regulating NETosis through the IL1β pathway (Yi et al. 2021).

Recent studies have revealed that neutrophils, previously considered as bystanders in the tumor microenvironment, also play an important role in tumor development, such as tumor metastasis and early recurrence, by producing NETs (Tohme et al. 2016; Yang et al. 2020; Lu et al. 2022). For instance, in our previous study, GSK-650,394 was involved in the generation of a premetastatic niche in the liver of colorectal cancer by inhibiting the SGK1/ERK/NETs pathway in hepatic neutrophils, thereby attenuating the progression of CRLM after IR (Li et al. 2023a, b). The hypoxic environment induced by IR triggers the production of HIF1α and accelerates hepatocellular carcinoma growth through the IL-6/JAK/STAT3 pathway (Hamaguchi et al. 2016). Cell dysfunction, proinflammatory factor production, and increased matrix metalloproteinases caused by hepatic IR injury can promote the development of metastatic tumors in the mouse liver (Nicoud et al. 2007). NETs interact with other immune cells in the tumor microenvironment in the liver to jointly regulate tumor recurrence and metastasis (Yang et al. 2020; Larco et al. 2004; Nakamura et al. 2019). In this study, we evaluated the role of GS-9973 in liver cancer by establishing a mouse model of colorectal cancer liver metastasis and hepatocellular carcinoma recurrence. The data indicated that preoperative and postoperative programmed administration of GS-9973 reduced CRLM and HCC recurrence in mice by inhibiting NETosis and the differentiation of liver Treg cells. Other studies have shown that treatment of MDSC stimulated by Candida tropicalis with the SYK small molecule inhibitor R406 resulted in a significant down-regulation of PKM2 activation and nuclear translocation, thereby weakening the immunosuppressive effect of MDSC (Zhang et al. 2022). Therefore, blocking SYK-induced NETosis is expected to be an effective means to improve liver IRI and reduce tumor recurrence and metastasis of liver tumors.

Reperfusion injury after partial hepatectomy and liver transplantation is still an unsolved problem in liver surgery. Our report fills the gap of the role of SYK in the inflammatory injury caused by liver IR. This contributes to a better understanding of the mechanism of postoperative liver inflammation, and further explores the therapeutic strategies to prevent postoperative liver dysfunction.

## Conclusions

In summary, our study demonstrated that SYK is activated in response to hepatic IRI. SYK directly or indirectly modulates the formation of NETs by regulating PKM2/P-STAT3 in neutrophils or NLRP3/IL-1β in macrophages. Therefore, the administration of small molecule inhibitors targeting SYK, including GS-9973, is a potential strategy for treating postoperative liver injury and tumor recurrence in patients undergoing hepatectomy.

## Electronic supplementary material

Below is the link to the electronic supplementary material.

Supplementary Material 1

## Data Availability

No datasets were generated or analysed during the current study.

## Abbreviations

SYK: Spleen tyrosine kinase
IRI: Ischemia reperfusion injury
NETs: Neutrophil extracellular trap
CRLM: Colorectal liver metastasis
sALT: Serum alanine aminotransferase
sAST: Serum aspartate aminotransferase
TUNEL: Terminal deoxynucleotidyl transferase-mediated deoxyuridine triphosphate nick-end labeling
