# Supplementary material for: SYK promotes the formation of neutrophil extracellular traps by inducing PKM2 nuclear translocation and promoting STAT3 phosphorylation to exacerbate hepatic ischemia-reperfusion injury and tumor recurrence
Source: Mol Med. 2024 Sep 11;30:146. doi: 10.1186/s10020-024-00907-7 (PMC11391729; doi:10.1186/s10020-024-00907-7)
Supplement: Supplementary file 1 — Supplementary Material 1 [file 10020_2024_907_MOESM1_ESM.docx]

**supplementary materials**


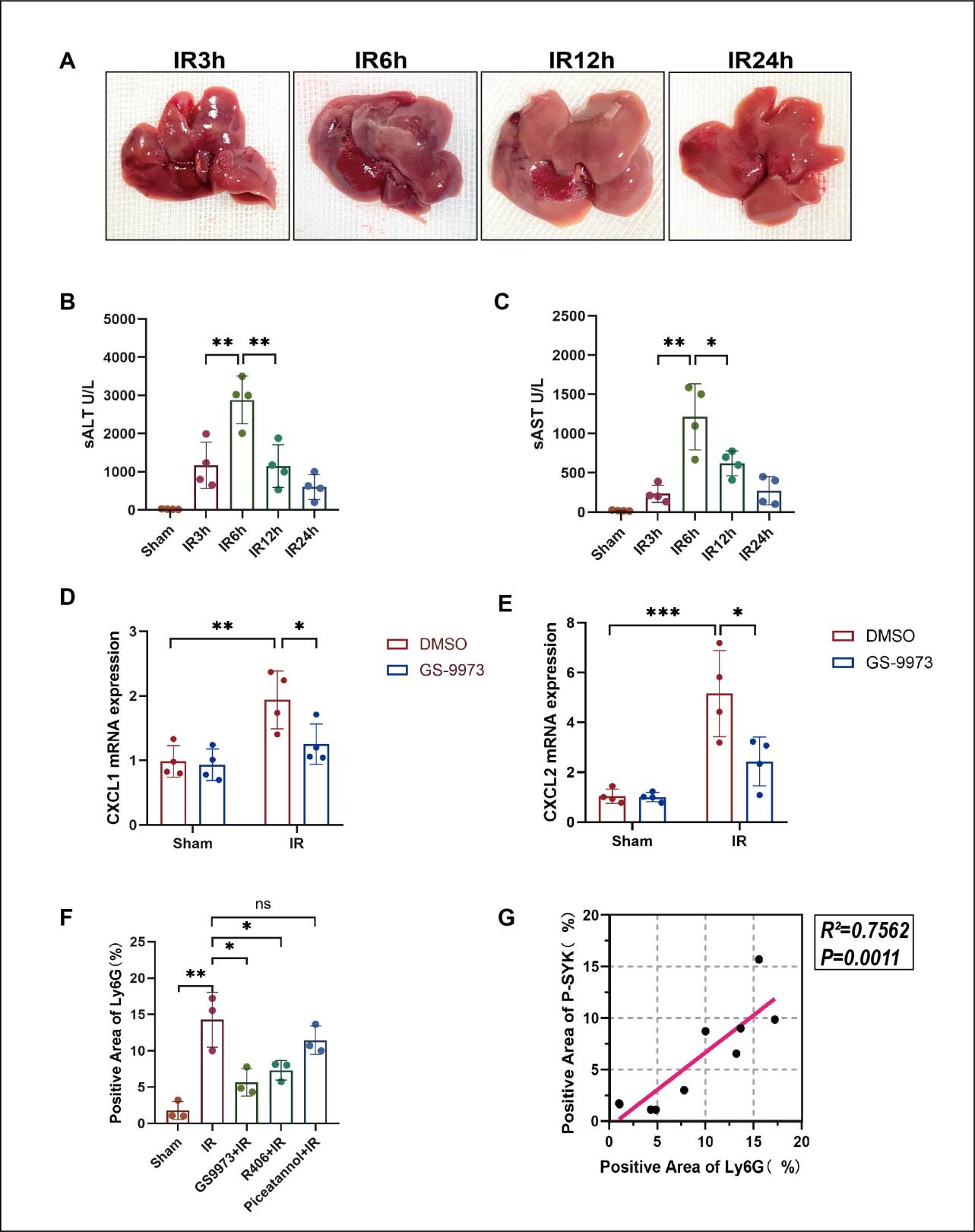


**Fig S1A-C** After 90 minutes of hepatic ischemia in mice, liver tissue was obtained at different time points after reperfusion to measure serum ALT and AST levels (n=4). **D,E** The mice were intraperitoneally injected with GS-9973 before operation, and the mRNA levels of CXCL1 and CXCL2 in liver tissues were detected by qPCR 6h after liver IR (n=4). **F** The expression of Ly6G in mouse liver sections was detected by immunohistochemistry (n=3). **G** Correlation analysis on the positive areas of P-SYK and Ly6G in immunohistochemistry (n=10). **p* < 0.05，***p* < 0.01，****p* < 0.001.


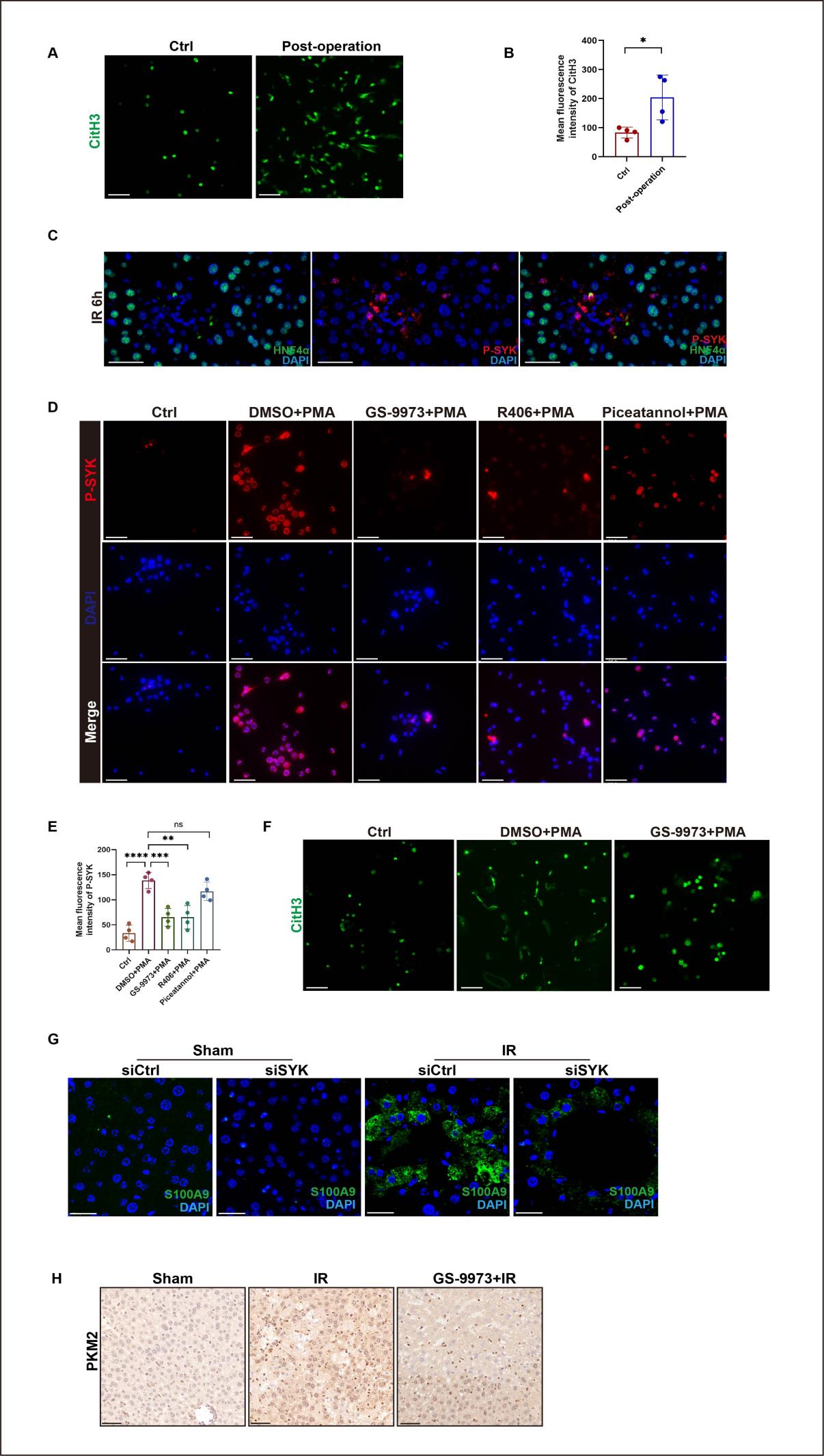


**Fig S2 A,B** Collect neutrophils of liver from mice underwent IR surgery, observe the expression of CitH3 under fluorescence microscopy, and quantify the fluorescence intensity. Scale bars, 50μm (n=4). **C** Immunofluorescence was conducted to detect the co-localization of P-SYK and HNF4α in the liver of mice after 6h of ischemia-reperfusion.Scale bars,40μm (n=4). **D,E** Murine bone marrow neutrophils were isolated, and the expression of P-SYK in response to PMA stimulation under the treatment of three SYK inhibitors was detected by fluorescence microscopy and quantified the fluorescence intensity. Scale bars, 50μm (n=4). **F** Neutrophils were isolated from peripheral blood of patients one day before surgery, pretreated with GS-9973 or DMSO for 2 hours, and then stimulated with PMA for 3h. The expression of CitH3 was detected by fluorescence microscopy. Scale bars, 50μm (n=4). **G** Mice were injected with a mixture of siSYK and mannose-conjugated polymer before IR, and liver tissues were extracted 6h after reperfusion. The expression of S100A9 was detected by fluorescence microscopy. **H** The expression of PKM2 was detected by Immunohistochemistry. **p* < 0.05，***p* < 0.01，****p* < 0.001,*****p* < 0.0001.


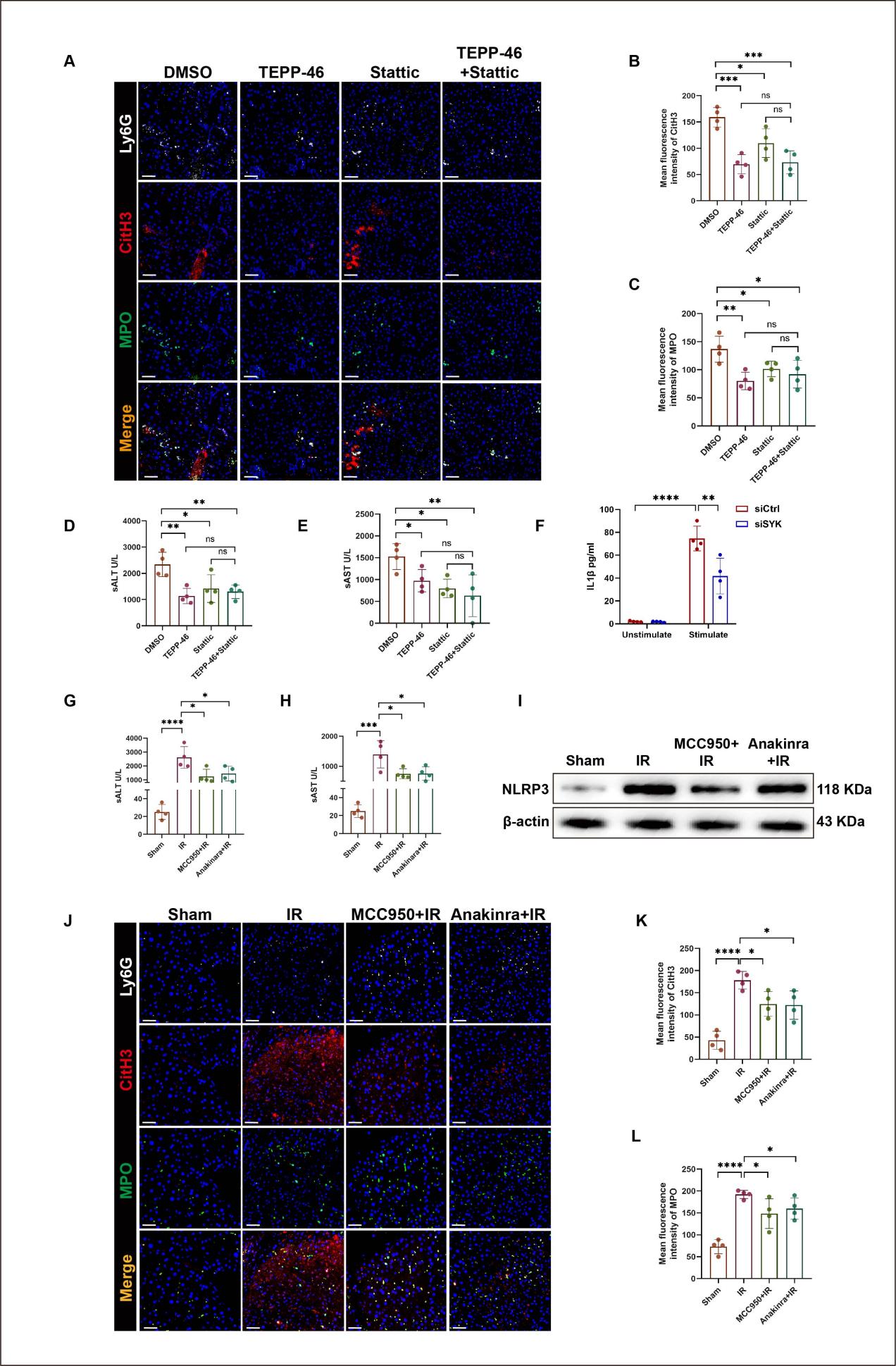


**FigS3 A** Mice were intraperitoneally injected with TEPP-46(30mg/kg) or Stattic(10mg/kg) before IR surgery, and liver tissue was obtained after 6 hours of reperfusion.The expression of Ly6G, CitH3, and MPO in liver tissue was detected by immunofluorescence. Scale bars, 20μm (n=4). **B, C** Quantification of the fluorescence intensity of CitH3 and MPO in figure A. **D, E** The levels of serum ALT and AST in mice (n=4). **F** Raw 264.7 cells were transfected with siSYK and stimulated with 1ug/ml LPS for 6h. ELISA was used to detect the level of IL1β in the culture medium (n=4). **G,H** The mice were intraperitoneally injected with MCC950(20mg/kg) and Anakinara(20mg/kg) before liver IR, and serum ALT and AST levels were measured 6 hours after reperfusion (n=4). **I** Western Blot was performed to detect the expression of NLRP3 in liver tissue (n=4). **J** Immunofluorescence staining was conducted to detect the expression of Ly6G, MPO, and CitH3 in the liver tissue of mice treated with MCC950 or Anakinra before surgery. Scale bars,20μm (n=4). **K, L** The expression levels of MPO and CitH3 were quantified (n=4). **p* < 0.05，***p* < 0.01，****p* < 0.001,*****p* < 0.0001.

***Primers(mice)***

| Gene | Forward | Reverse |
| --- | --- | --- |
| SYK | TCTTACAAGCCAGATGGGCTA | GGCTTTGGGAAGGAGTAGGA |
| TNFα | GGTGCCTATGTCTCAGCCTCTT | GCCATAGAACTGATGAGAGGGAG |
| IL1β | TGGACCTTCCAGGATGAGGACA | GTTCATCTCGGAGCCTGTAGTG |
| IL10 | CGGGAAGACAATAACTGCACCC | CGGTTAGCAGTATGTTGTCCAGC |
| CXCL1 | TCCAGAGCTTGAAGGTGTTGCC | AACCAAGGGAGCTTCAGGGTCA |
| CXCL2 | CATCCAGAGCTTGAGTGTGACG | GGCTTCAGGGTCAAGGCAAACT |
| IL6 | TACCACTTCACAAGTCGGAGGC | CTGCAAGTGCATCATCGTTGTTC |
| TGFβ | TGATACGCCTGAGTGGCTGTCT | CACAAGAGCAGTGAGCGCTGAA |
| β-actin | GGAGATTACTGCCCTGGCTCCTA | GACTCATCGTACTCCTGCTTGCTG |
